# MRI-based torsion measurement of the lower limb is a reliable and valid alternative for CT measurement: a prospective study

**DOI:** 10.1007/s00167-023-07533-6

**Published:** 2023-08-17

**Authors:** Leonard Grünwald, Tina Histing, Fabian Springer, Gabriel Keller

**Affiliations:** 1 Department of Diagnostic and Interventional Radiology, University Hospital Tübingen, Eberhard Karls University Tübingen, 72076 Tübingen, Germany; 2 Department of Traumatology and Reconstructive Surgery, BG Trauma Center Tübingen, Eberhard Karls University of Tübingen, Schnarrenbergstrasse 95, 72076 Tübingen, Germany

**Keywords:** Torsional malalignment, Tibia, Femur, CT, MRI, Reliability, Validity, Waidelich, Radiation exposure

## Abstract

**Purpose:**

The aim of this study was to compare MRI-based torsion measurements of the lower limb to a well-established CT-based assessment in a prospective inter- and intraindividual approach.

**Methods:**

A total of 26 patients (age 28.8 years ± 11.0) were enrolled beginning in January 2021 until August 2022. Inclusion criteria were the clinical indication for torsion measurement of the lower limb. CT and MRI imaging were performed with a standard operating procedure, to ensure that all patients were examined in a standardized position. The examinations were planned on a coronal scout view based on prominent anatomical landmarks. Femoral and tibial torsion were measured individually. Torsion measurements were analysed twice: immediately after examination and after 3 weeks. Subsequently, intra-rater and parallel test reliability was calculated accordingly.

**Results:**

High significant results for CT and MRI measurements for both tibia (MRI: *r* = 0.961; *p* ≤ 0.001; CT: *r* = 0.963; *p* ≤ 0.001) and femur (MRI: *r* = 0.980; *p* ≤ 0.001; CT: *r* = 0.979; *p* ≤ 0.001) were obtained by calculated intra-rater reliability, showing that measurements were highly consistent for MRI and CT, respectively. Parallel test reliability for time point 1 as well as time point 2 was also highly significant and ranged from *r* = 0.947 to *r* = 0.972 (all with *p* ≤ 0.001, respectively) for both tibia and femur, showing a high concordance between the two measurements.

**Conclusion:**

Measurement of tibial as well as femoral torsion was comparable for CT and MRI measurement. Therefore, this study supports MRI measurement as an equivalent alternative for CT measurement concerning torsional malalignment to reduce exposure to radiation.

**Level of evidence:**

Level II.

## Introduction

Torsional malalignment has gained increasing interest in clinical practice as well as in research [5, 7, 8]. Several clinical and experimental studies showed that deviations from normal torsional alignment—whether congenital or post-traumatic—play a major role for the biomechanics of the knee joint [6, 16]. It is well known that torsional malalignment has great influence on the development of osteoarthritis as well as in patellofemoral disorders [1, 7, 14].

Gold standard for the assessment of lower limb malalignment in the transversal plane is computed tomography (CT) with transversal reconstructions. In comparison to anatomic measurement (for example in cadaveric studies), CT is the most precise and shows best reproducibility [1, 6, 21]. To perform the measurement, the CT scan should include the ankle region, the knee region and the hip region [11]. This results in a not negligible exposure to ionizing radiation [2]. CT diagnostics are recommended for a vast number of different disorders: for example, in patients with fractures of the femur or patellar dislocations, common guidelines recommend an assessment of lower limb malalignment conducted by CT [13, 19]. Completely avoiding ionizing radiation, however, is to be preferred for the—mostly younger—patient collective.

To our knowledge, there are no prospective studies with modern scanners to compare assessment of lower limb malalignment between CT and MRI torsion measurement. Only studies referring to tibial or femoral measurement separately are accessible or—if both were measured—patients did not receive MRI and CT measurements concurrently [12]. Therefore, the purpose of this study was to compare an MRI-based protocol for torsion measurement of the lower limb to a well-established CT-based assessment in a prospective approach and with a sensibly defined standardized flow trace for each MRI and CT conduction that ensures the same supine position and same processes. The hypothesis of this study was that MRI assessment of femoral and tibial torsion according to the Waidelich method [20] achieves equivalent results to CT assessment.

## Materials and methods

Institutional review board approval about all aspects of the study from an ethical and legal point of view was obtained (04.11.2020, IRB number: 780/2020BO1).

The study was conducted in accordance with the Declaration of Helsinki (as revised in 2013).

This study was prospectively registered on the German Clinical Trials Register (No.DRKS00023178). Inclusion criteria were the clinical indications for torsion measurement of the lower limb, which included patients with patella dislocation, anterior knee pain and clinical suspicion of torsional malalignment. Exclusion criteria were age under 18 years, the presence of metal implants at the lower limbs and the lack of written informed consent. Accordingly, in the period of January 2021 until August 2022, *n* = 26 patients could be enrolled.

The following demographic parameters were recorded: age at time of examination, gender and self-reported body mass index (BMI). CT and MRI were conducted on the same day.

### Technical parameters of the CT image acquisition for torsion measurement of the lower limb

**Fig. 1:**
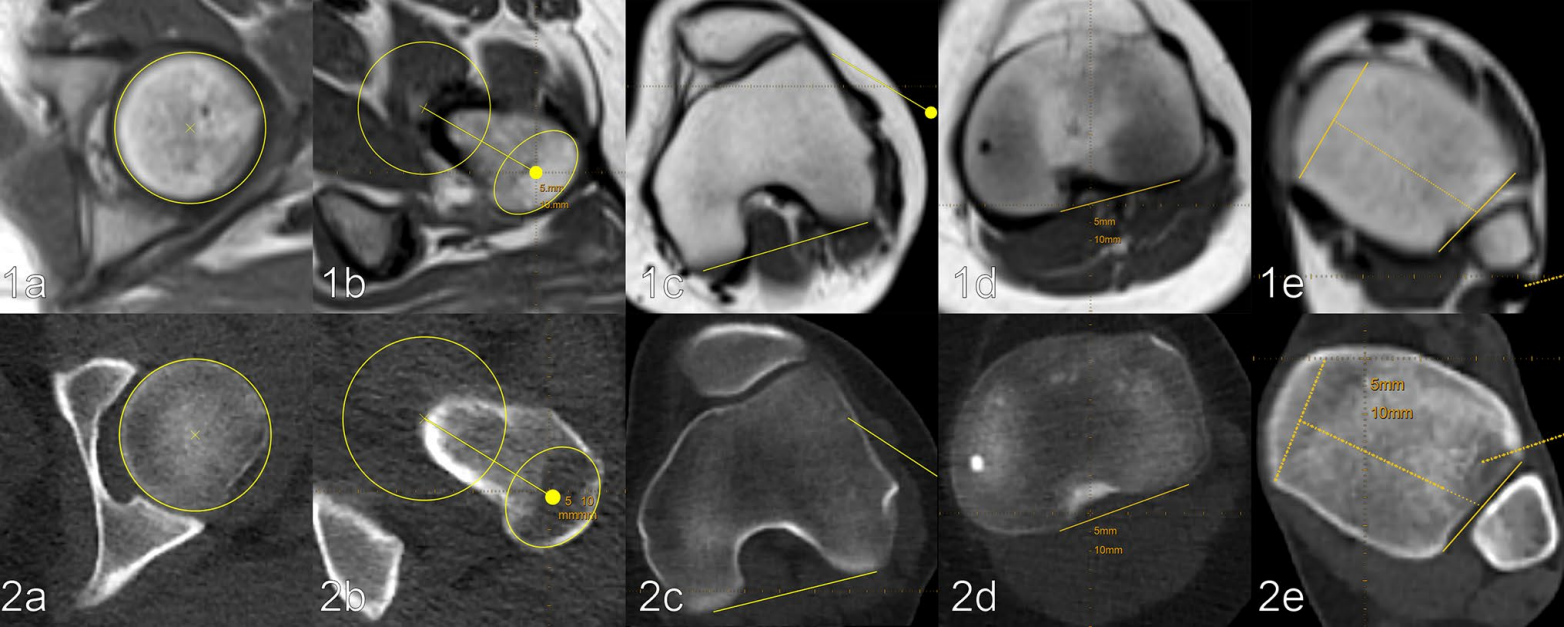
Comparison of CT and MRI for torsion measurement of the lower limb in an exemplary patient (female, 18 years, BMI = 24 kg/m^2^), scanned in the same position. The figure depicts exemplarily the captured images as well as the drawn reference lines. Number (1) depicts MRI images and number (2) CT images, respectively, for: **a** hip centre, **b** centre of the greater trochanter, **c** posterior margin of the femoral condyles, **d** posterior margin of the tibial plateau, **e** line through the centre of the medial malleolus and centre of the fibular incisura of the tibia

CT image acquisition was performed using a 128-slice, single source CT (SOMATOM Definition Edge, Siemens Healthineers, Forchheim, Germany) using a previously evaluated and thereafter established ultralow dose protocol with a pitch of 1.0, rotation time of 0.5 s, collimation of 128 × 0.6 mm and a scan time of 2.21 s (hip), 2.11 s (knee) and 1.77 s (ankle) [8]. An automated tube current modulation (CARE Dose4D, Siemens Healthineers, Forchheim, Germany) was used for all regions (hip, knee, ankle). Furthermore, an automated tube voltage selection (CARE kV, Siemens Healthineers, Forchheim, Germany) was additionally used for all regions and was set to optimize tube current for the depiction of osseous structures. Reference settings were set as follows: hip (100 kV, 20 mAs), knee (80 kV, 20 mAs) and ankle (80 kV, 10 mAs). Furthermore, raw data-based iterative image reconstruction (SAFIRE—Sinogram Affirmed Iterative Reconstruction, Siemens Healthineers, Forchheim, Germany) was used at strength 3 for all regions. Image reconstruction was performed using a medium sharp kernel, a 3 mm slice thickness and a bone window (centre/width: 450HU/1500HU). According to the institutional standard operating procedures, all patients were examined in a standardized supine position, feet first and the feet secured together. The examinations were planned on a coronal scout view based on prominent anatomical landmarks: at the hip, top of the femoral head to the upper margin of the lesser trochanter; at the knee, top of the patella to the middle of the fibular head; and at the ankle, 2 cm above the tibial plafond to the tip of the medial ankle (Fig. 1).

### Technical parameters of the MR image acquisition for torsion measurement of the lower limbs

MR image acquisition was performed on a 1.5 T scanner (MAGNETOM Area, Siemens Healthineers, Forchheim, Germany) using solely the coils integrated in the MR scanner and the patient table. The study protocol consisted of three slice blocks (hip, knee, ankle) of T1-weighted turbo spin echo sequences in transversal orientation. Repetition time was set to 578 ms, echo time to 12 ms, flip angle to 180°, turbo factor to 3, parallel acquisition techniques to 2, number of averages to 1 and number of concatenations to 2, resulting in an acquired voxel size of 1.0 × 1.0 × 6.0 mm^3^ and an acquisition time of 1:07 min (Fig. 1). As during the CT, all patients examined in standardized supine position, feet first and the feet secured together, MRI was planned on a coronal scout view in the same manner (Fig. 1).

### Evaluation of the lower limb torsion in MRI and in CT

MRI and CT torsion measurements were performed on pseudonymized examinations and in randomized order (using the SPSS random number generator feature). Femoral and tibial torsion were measured separately according to Waidelich [20]. The Waidelich method was chosen due to its good reproducibility in CT measurements and due to existing standard values [17]. In accordance with the method, femoral neck antetorsion was measured as the angle between a line central through the femoral head and central through an ellipse of the greater trochanter and a second line along the posterior margin of the femoral condyles. Tibial torsion was measured between a line along the posterior margin of the tibial plateau and a line central through the tibial and fibular parts of the ankle joint. Torsion measurements were analysed twice by the same orthopaedic specialist (8 years of experience in lower limb torsion measurements and fellowship trained)—immediately after the procedure and again after three weeks. An experienced radiologist additionally analysed a total *N* = 40 lower limbs to evaluate inter-rater reliability. For torsion measurement, the software package mediCAD 3D Knee Version 2.5 (Hectec, Landshut, Germany) was used.

### Statistical analysis

For statistical analyses, IBM SPSS Statistics for Windows, version 23.0 (IBM Corp., Armonk, N.Y., USA), was used. Analysed were the intra-rater reliability, inter-rater reliability and the parallel test reliability. Bland–Altman plots were constructed. For reliabilities, the Pearson product–moment correlation coefficient was used. A reliability coefficient higher than 0.80 is interpreted to be “good” and a coefficient higher than 0.90 is interpreted to be “high” according to Danner [4]. The level of significance was set at *α* ≤ 0.05. In planning the test for the establishment of equivalence between the MRI-based and CT-based measurements using a Bland–Altman analysis, it was determined that at a Type I error rate of *α* = 0.01, a Type II error rate of *β* = 0.10 (i.e. Power = 0.90), assuming a bias of 0 and a correlation of *r* = 0.995 between the measurements, that both show variability of 1.41508 (preconditions found in a previous study) the differences between the measurements can be detected to be less than ± 5° with *N* = 25 independent observations [10]. Further sensibly assuming that the correlation of measurements for two different limbs within patients is higher than the correlation of limbs from different patients, the power will be higher (everything else being equal).

## Results

The total study sample consists of *N* = 52 examined lower limbs, derived from *N* = 26 patients with an age range between 18 and 61 years (*M* = 28.8 ± 11.0). An equal number of men (*N* = 13) and women (*N* = 13) took part in the study. BMI ranged from 20.2 to 43.4 kg/m^2^ (*M* = 28.3 kg/m^2^ ± 6.6 kg/m^2^).

For all 52 lower limbs, descriptive data are displayed in Table 1 for all time points and measurements in CT and MRI.

**Table 1:** Measurement for all *N* = 52 lower limbs for the femur and tibia at both time points and CT as well as MRI (*M* = mean value; SD = standard deviation)

Time point	Femur				Tibia			
	1		2		1		2	
Imaging	CT	MRI	CT	MRI	CT	MRI	CT	MRI
Minimum	− 58.5	− 63.7	− 56.0	− 57.7	15.3	15.9	16.0	15.9
Maximum	− 9.1	− 8.6	− 7.9	− 8.0	56.3	58.1	56.7	55.1
*M*	32.6	− 32.5	− 31.9	32.2	36.9	37.6	36.9	37.1
SD	11.6	11.9	11.4	11.5	9.1	9.2	9.2	9.0

First, intra-rater reliability was tested for tibia and femur for measurement with MRI as well as CT. Results were *r* = 0.961, *p* ≤ 0.001 (MRI) and *r* = 0.963, *p* ≤ 0.001 (CT) for tibial measurements and *r* = 0.980, *p* ≤ 0.001 (MRI) as well as *r* = 0.979, *p* ≤ 0.001 (CT) for femoral measurements. These highly significant results indicate that measurements are highly consistent for MRI as well as CT. Therefore, measurement for both imaging techniques proved to be highly consistent for different time points (Table 2).

**Table 2:** Intra-rater correlation for tibia and femur for both methods (*r* = Pearson correlation coefficient; *p* = significance)

	Correlation (r) between measurement 1 and 2
Femur	
MRI	*r* = 0.980** *p* ≤ 0.001
CT	*r* = 0.979** *p* ≤ 0.001*
Tibia	
MRI	*r* = 0.961** *p* ≤ 0.001
CT	*r* = 0.963** *p* ≤ 0.001

Parallel test reliability was calculated for each femur and tibia, for both time points 1 and 2 for the comparison of CT and MRI. Correlation coefficients for femoral measurement ranged from *r* = 0.972, *p* ≤ 0.001 (time point 1) to *r* = 0.965, *p* ≤ 0.001 (time point 2) and were highly significant. For tibial measurements correlation coefficients ranged from *r* = 0.947, *p* ≤ 0.001 (time point 1) to *r* = 0.948, *p* ≤ 0.001 (time point 2) and were also highly significant. Accordingly, for both tibia and femur, parallel test reliability can be interpreted to be “high”. All results are presented in Table 3.

**Table 3:** Parallel test reliability of measurement of the femur and tibia, for time points 1 and 2 (*r* = Pearson correlation coefficient; *p* = significance; Min = minimum; Max = maximum; *M* = mean value; SD = standard deviation)

Time point	Femur		Tibia	
	1	2	1	2
Correlation	*r* = 0.972** *p* ≤ 0.001	*r* = 0.965** *p* ≤ 0.001	*r* = 0.947** *p* ≤ 0.001	*r* = 0.948** *p* ≤ 0.001
Min–max of differences	0.0–9.3	0.0–9.7	0.1–8.2	0.1–8.3
*M* ± SD for differences	2.1 ± 1.9	2.2 ± 2.1	2.2 ± 2.1	2.4 ± 1.8

**Fig. 2:**
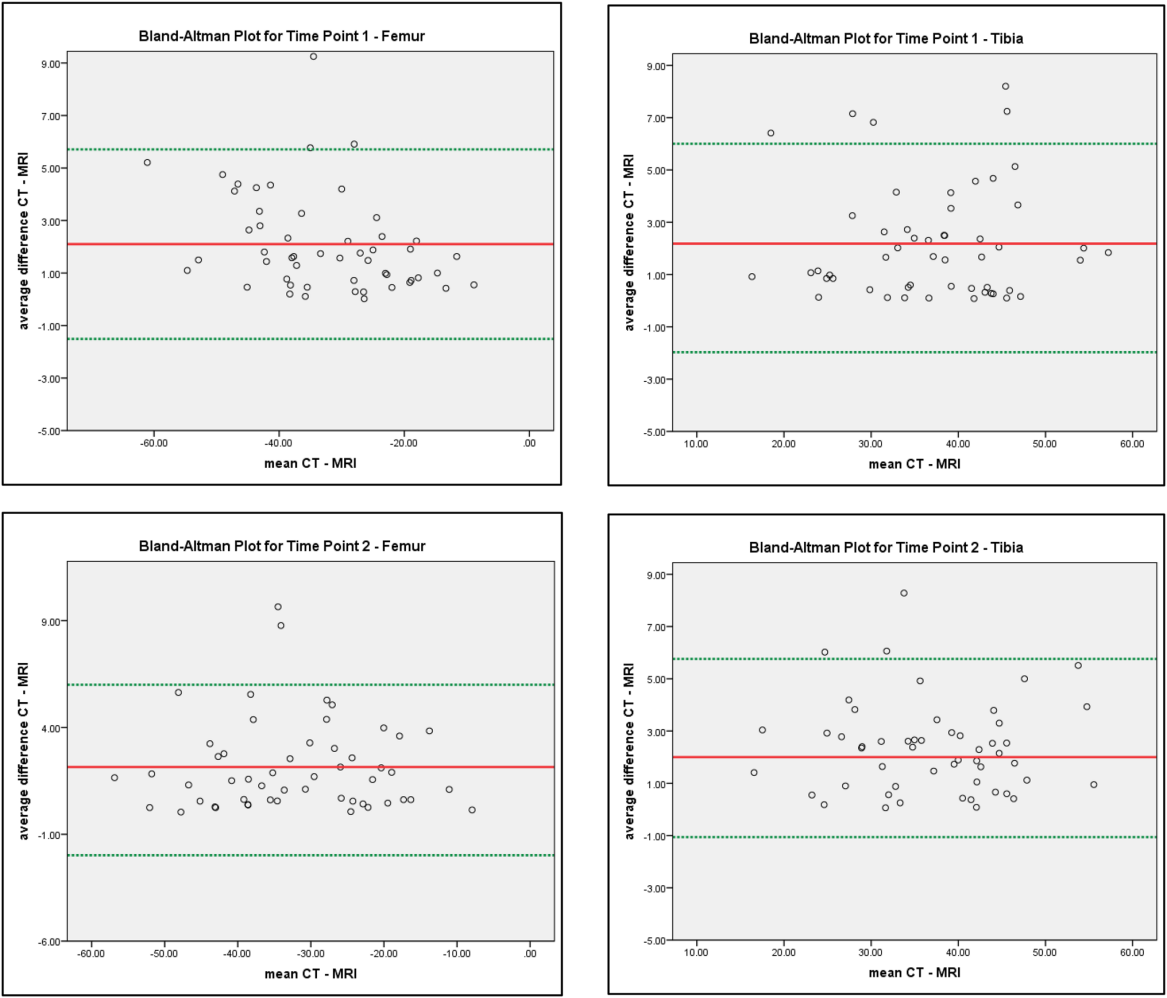
Bland–Altman plot for tibia (right) and femur (left) at time points 1 (above) and 2 (below). The Bland–Altman plots depicts the correspondence of the imaging techniques. Red lines mark the respective mean value, and green lines mark the standard deviation in accordance with the mean value

To continue and develop the results of the parallel test reliability further, Bland–Altman plots were generated. Average differences for measurements of MRI and CT were calculated for time points 1 and 2 to check for potential distortions. Bland–Altman plots indicated a small bias of about 2° for femoral as well as for tibial measurements, as well as a small number of outliers. Analysis showed that these outliers were due to difficult patient positioning for patients with high BMI or due to measurement error (1 case). The plots are displayed in Fig. 2.

To further extend and strengthen the previous results, inter-rater reliability was calculated with measurements made by an experienced radiologist, who analysed a random selection of *N* = 40 lower limbs. Calculated inter-rater reliability for CT was *r* = 0.876; *p* ≤ 0.001 for femoral and *r* = 0.830, *p* ≤ 0.001 for tibial measurements.

For MRI inter-rater reliability was *r* = 0.883, *p* ≤ 0.001 for femoral measurements and *r* = 0.909, *p* ≤ 0.001 for tibial measurements.

Therefore, inter-rater reliability can be interpreted as high for both measurement methods, with MRI to show slightly better values than CT in comparison.

## Discussion

The most important finding of the study was that calculated parallel test reliability for time point 1 as well as time point 2 was in ranges of *r* = 0.947 to *r* = 0.972 for both tibia and femur. For outliers, we identified one measurement error; the others had been patients with high BMI, causing difficult circumstances in patient positioning. As this problem is commonly seen in clinical practice, we decided to include these cases, to enable the study to give a realistic depiction. The results are strengthened by the high intra-rater and inter-rater reliability, showing that measurements were highly consistent for MRI as well as CT. This is the precondition for stable and valid parallel test reliability testing. These findings constrain a high agreement of MRI and CT torsion measurements of the lower limb. In contrast to these results, Botser et al. report a systematic bias for measurement of the femur of − 8.9° for MRI in comparison to CT. However, the study had a retrospective design and it was assumed that different patient positioning during MRI led to the discrepancies in results [3]. The problem of different patient positioning was addressed in this study with a standard operating procedure, which ensured that all patients were examined in a standardized position (supine, with feet first, and the feet secured together). This might explain why deviations between MRI and CT in this study are relatively small. Nevertheless, we detected a small bias of approximately 2° between CT and MRI measurement. For using torsional measurements to plan corrective osteotomies, a bias of 2° is acceptable, as osteotomies usually have a tolerance of at least 5° for deviations of the desired angle. Therefore, this bias is not critical for patient management. However, in particular for the comparison of differences between torsional measurements of the right and the left lower limb, this possible systematic bias warrants dedicated further research, most suitable in further prospective studies with standardized patient positioning protocol. If this bias of 2° would be confirmed in further studies, it would be reasonable to develop an agreed mathematical formula that enables clinicians to compare MRI and CT measurements on a solid foundation. Additionally, to further information, all examinations were planned on a coronal scout view based on prominent anatomical landmarks. This is in line with a study of Muhamad et al. that addressed the question of different landmarks in measuring femoral and tibial torsion and assessed 62 patients between 7 and 19 years [12]. Their results showed that MRI is comparable to CT concerning reproducibility, if similar landmarks are used. However, the nature of the study was retrospective and no patient enrolled had MRI and CT simultaneously to evaluate torsion. Schmaranzer et al. referred more detailed to the question of landmark setting in the assessment of femoral torsion to compare MRI and CT measurement [15]. They retrospectively compared four different measurement methods (regarding landmark levels) in 57 hips. Their result was that MRI- and CT-based femoral torsion measurement showed high agreement and comparable reliability and reproducibility, depending on the level of selected landmarks used to define the proximal reference axis. Concerning comparison with true torsion of the femur, Beebe et al. performed a cadaveric study with 12 femora [1]. They found that CT-axial was the most accurate and reproducible measurement when compared with true torsion of the femur, but closely followed by MRI-axial. They propose using MR-axial images in clinical situations where radiation exposure needs to be limited. These results and the proposal are reflected by the study of Tomczak et al., who compared CT and MRI measurement in 19 children and 25 adults [18]. MRI allowed precise anatomic measurements and produced reliable and reproducible results. They recommend MRI for preoperative planning in paediatric patients with femoral rotation osteotomies. For CT assessment, Recent studies showed that a median 96% decrease of radiation exposure compared to a standard protocol resulted in a continuously high rated image quality [8].

As a limitation of the presented results, the relatively small sample size of *n* = 26 has to be mentioned, although the required sample size was overachieved according to the power calculation prospectively registered in the German Clinical Trials Register (No. DRKS00023178). Bland–Altman plots indicated a bias of about 2° for femoral as well as for tibial measurements. Although this small difference seems not critical for patient management, in particular for the comparison of differences between torsional measurements of the right and the left lower limb, this possibly systematic bias warrants dedicated further research. Moreover, it has to be named that individual cases showed a discrepancy of the measured angles on MRI and CT of more than 8°. Nevertheless, evaluated across the entire collective, the comparison of MRI and CT torsion measurements showed a high level of agreement, which suggests that both MRI and CT are valid for torsion measurements of the lower limb. This is important especially for radiation-sensitive patients such as young people. However, in populations that are not that radiation sensitive, it is arguable whether CT might be preferable for torsion measurement of the lower limb. Concerning measurement methods, it also has to be stated that only the Waidelich method was chosen to evaluate lower limb torsion, due to its good reproducibility in CT measurements and due to existing standard values. However, for cases with a very flat incisura fibularis, the creation of the distal line for measurement of the tibial torsion might be complicated. Most importantly, as specific measurement methods depend on varying anatomic landmarks, it is of utmost importance to name the used measurement method to guarantee comparability of results. Also, as a limitation it has to be mentioned that patients with metal implants were excluded. Metal implants might be a contraindication for MRI and metal artefacts might compromise MRI torsion measurements more than CT torsion measurements. For CT measurement, lately a study showed feasibility of an ultralow dose CT in patients with metal implants [9]. Based on the results of the current study, it is now important to further promote research for specific patient collectives, namely with metal implants, if MRI measurement can prove to be an equivalent alternative in these cases.

In the light of the preceding studies, the results of this study have high impact for measurement of torsional malalignment, as—to our knowledge—it is the first prospective study to check for comparability between MRI and CT. With all patients receiving MRI and CT imaging on the same day, but more crucially following the same standard operating procedure and patient positioning, and therefore the same preconditions had been implemented for each measurement. Additionally, this study did not only address femoral, but also tibial torsion measurement, which has been rare in foregoing studies. Therefore, this study provides valuable information not only for surgeons, but also radiologists or scientists, who need to assess torsional malalignment. Especially in these cases, when imaging has to be conducted several times and patients are of younger age, MRI measurement techniques seem to be a worthy and reliable alternative.

## Conclusion

Measurement of tibial as well as femoral torsion is comparable for CT and MRI measurement. Therefore, this study supports MRI measurement as a valid alternative for CT measurement for torsional malalignment, especially in cases with high importance to reduce radiation exposure.

## Data Availability

The data that support the findings of this study are available from the corresponding author GL, upon reasonable request.
